# Insect Diversity in *Pinus sylvestris* Forest Stands Damaged by *Lymantria monacha*

**DOI:** 10.3390/insects15030200

**Published:** 2024-03-17

**Authors:** Vytautas Čėsna, Artūras Gedminas, Jūratė Lynikienė, Diana Marčiulynienė

**Affiliations:** Institute of Forestry, Lithuanian Research Centre for Agriculture and Forestry, Liepų 1, LT-53101 Girionys, Lithuania; arturas.gedminas@lammc.lt (A.G.); jurate.lynikiene@lammc.lt (J.L.); diana.marciulyniene@lammc.lt (D.M.)

**Keywords:** Scots pine, nun moth, pest outbreaks, biological control, *Bacillus thurningiensis*

## Abstract

**Simple Summary:**

The nun moth (*Lymantria monacha* L.), a defoliator of conifers and broad-leaved trees, is expanding its range, and outbreaks are increasingly occurring in the forests of central and eastern Europe. The only way to control and eradicate mass outbreaks of the pest is aerial spraying with a biological insecticide, Foray 76B. However, knowledge of variations in non-target insect assemblages following a treatment is limited. The present study aimed to determine the effect of the treatment on the diversity of non-target insects in *Pinus sylvestris* stands in three regions of Lithuania in the year following a nun moth outbreak. The treatment was found to influence the diversity of psyllids, ants, and beetles. Treated pine forests near the Baltic Sea exhibited lower insect species richness and a decreased relative abundance of beetles on the forest floor. The spraying influenced a reduction in the relative abundance of *Carabus arcensis* in the forests in the southern part of the country. The treatment also influenced the movement of ants from the tree canopy to the forest floor at all studied locations.

**Abstract:**

Outbreaks of *Lymantria monacha* are of great concern, as their occurrence is predicted to become more intense and frequent due to a warming climate. A frequent treatment to control mass outbreaks of the pest is with the bioinsecticide Foray 76B. However, knowledge of how this treatment affects non-target insect species is limited. We surveyed the assemblages of non-target epigeal and arboreal insects in *Pinus sylvestris* forests in the year following bioinsecticide application. A collection of insects using sweep nets and pitfall traps was carried out in *L. monacha*-infested pine stands, (i) treated with Foray 76B and (ii) untreated, in three regions of Lithuania from May to October 2021. The results revealed that, in Neringa forests, species richness of the epigeal insects was lower in treated than in untreated sampling plots, with 36 and 41 different insect species, respectively. The relative abundance of epigeal Coleoptera in treated plots was 3.6%, while in untreated it was 53.2%. There was a significant decrease in the relative abundance of *Carabus arcencis* in Kapčiamiestis (by 7.4%) and Marcinkonys (by 16.7%). Treated plots were distinguished by lower relative abundance of arboreal Hymenoptera at all three study locations.

## 1. Introduction

Due to the warming climate, coniferous forests are increasingly vulnerable to various biotic and abiotic disturbances [1,2,3,4], including outbreaks of forest insects [5,6,7]. In consideration of its adaptability to a diverse range of soil and climatic conditions, Scots pine (*Pinus sylvestris* L.) is one of the most dominant coniferous tree species in hemiboreal forests across the northern hemisphere [8,9]. In Lithuania, Scots pine stands cover 34.5% of the total forest area [10]. 

Insects play a critical role in plant reproduction, soil fertility, forest health, and food-web interactions [11,12]. Most forest insect species are embedded in complex food webs [13]. Although some species of insects themselves provide food for birds, reptiles, bats, amphibians, and other animals [14], many of these species are predators that are important, especially during outbreaks of insect pests [15,16]. Specific insect species, such as beetles (Coleoptera) and ants (Formicidae), are indicators of forest health [17,18,19]. 

The nun moth (*Lymantria monacha* L.) is an important pest of Scots pine forests in central and northern Europe [20,21], expanding its distribution northward and causing frequent outbreaks [22]. Forests in Poland, the Czech Republic, and Germany [20,23], as well as in Lithuania and Latvia [24,25] experience frequent nun moth outbreaks. Since Scots pine stands provide habitats for many organisms, the spread of *L. monacha* can reduce their abundance or even threaten some species [26]. The nun moth outbreaks in Lithuania in 2018–2020 affected more than 6000 hectares of *P. sylvestris* stands [24]. In such extreme cases, *Bacillus thuringiensis* subspecies *kurstaki* Strain ABTS-351 (abbr. *Btk*, or Foray 76B), as an aerial treatment, is the most common biological agent used [27]. Although *Btk* is considered toxic only to lepidopterans (moths and butterflies) [28,29,30,31], the use of bioinsecticides utilizing *Bacillus thuringiensis* spores and toxins (including *Btk*) may impact non-target species [32]. These encompass beneficial insects involved in biological control [33], pollinators [34], and species coexisting with *Btk*-targeted insect pests [35]. However, there is a lack of knowledge regarding the indirect effects of treatment with Foray 76B against *L. monacha* outbreaks on such non-target species. To better understand and predict the effects of *L. monacha* outbreak treatments, a comprehensive assessment of their population dynamics is necessary. 

We hypothesized that treatment with Foray 76B to control *L. monacha* outbreaks in *P. sylvestris* stands can lead to significant changes in epigeal and arboreal insect abundance and species assemblages. The present study aimed to determine the diversity and abundance of epigeal and arboreal insect species in Scots pine forests following *L. monacha* outbreaks and treatment with bioinsecticide. 

## 2. Materials and Methods

### 2.1. Description of Forest Stands

**Figure 1:**
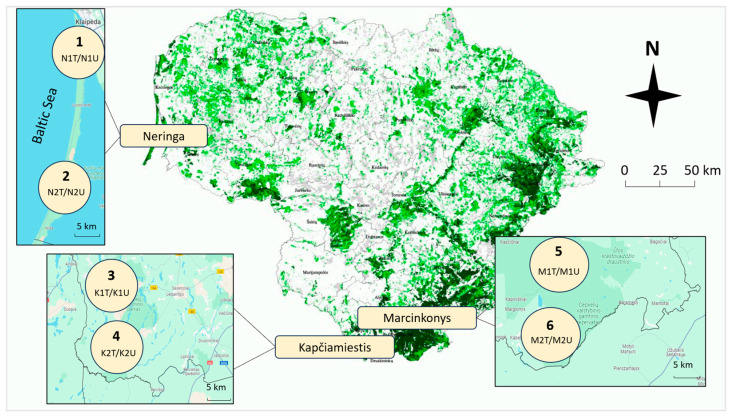
Map of Lithuania showing six *P. sylvestris* forest stands (1–6) damaged by *L. monacha* outbreaks—in Neringa, Kapčiamiestis, and Marcinkonys. Circles represent different forest stands, where samples were collected from treated (e.g., N1T) and untreated (e.g., N1U) plots.

Six 60–120-year-old *P. sylvestris* forest stands damaged by *L. monacha* outbreaks (tree crown defoliation, 30–60%) in Neringa, Kapčiamiestis, and Marcinkonys were selected for the study (Figure 1). Each of the six forest stands was treated with the biological insecticide Foray 76B in 2020 under the guidance of the State Forest Service. Meanwhile, the nearest *L. monacha*-damaged sampling plots (abbr. plots), which were not treated due to nearby protection zones, were selected as controls. The distance between the two forest stands at the same location was 5–30 km, and there was 2–3 km between treated and untreated plots of the same forest stand. The pine stands of Neringa grow in the dunes by the Baltic Sea at the transition between terrestrial and marine environments and have mild climatic conditions [36], whereas Kapčiamiestis and Marcinkonys in the southern part of the country experience harsher climatic conditions [37]. All the forest stands were characterized by normal humidity (N), very poor (a) or poor (b) fertility, light soil texture (l), and either cladoniosum (cl) or vaccinio-myrtilliosum (vm) vegetation type (Table 1).

In each of the plots, the trapping of epigeal and arboreal insects was performed using two different methods: (I) pitfall traps and (II) entomological sweep nets. 

### 2.2. Assessment of Epigeal Insects

Epigeal insects were sampled during the period of maximum arthropod activity in May–October 2021 (the following year after *L. monacha* outbreaks), using a modified Kamonen et al. (2015) method [39]. Pitfall traps, made of small plastic cups (6.5 cm diameter, 10 cm in depth), were dug into the substrate until they were flushed with the surrounding surface and filled with approximately 50 mL of 70% isopropyl alcohol. A nail-supported roof was installed 5 cm above the trap to reduce flooding and the accumulation of debris. In each plot, 5 traps were installed every 10 m, with a total of 30 traps in treated and 30 in untreated *P. sylvestris* plots. Insects were collected once a month, and the traps were filled with fresh isopropyl alcohol. The samples from 5 traps per plot were combined (total *n* = 12), transported to the laboratory, and dried at room temperature for 20 days. There was a total of 5 sample replicates over 5 months (6 forest stands × 2 plots per stand × 5 times). Identification of epigeal insects was performed using a Zeiss Stemi 2000-C microscope (Oberkochen, Germany) based on morphological characteristics and standard identification keys [40,41,42,43,44].

### 2.3. Assessment of Arboreal Insects

Sampling of arboreal insects was carried out during the same period and at the same plots as for epigeal insects (see above). In each plot, 50 sweeps around approximately 20 *P. sylvestris* tree branches were made with an entomological net in a 30 m^2^ crown area. Sampling was carried out on dry days. Collected arboreal insects were placed into a glass container with cotton wool, soaked in 99.2% chloroform for 15 min., sieved to remove plant material, and transferred to plastic boxes (*n* = 12). There was a total of 6 sample replicates over 6 monthly accountings (6 forest stands × 2 plots per stand × 6 times). The subsequent procedure for sample transportation, insect preparation, and identification was the same as described above for the epigeal insects. 

### 2.4. Statistical Analysis

PC-Ord 6 was used to calculate Shannon’s [45] diversity index. R (Version 4.2.1) with RStudio (Version 1.1.456) was used to calculate the following: (1) the number of insect individuals; (2) the relative abundance of insects; (3) the insect species richness; (4) the nonparametric chi-square test; (5) the nonparametric Mann–Whitney test; (6) the nonmetric multidimensional scaling (NMDS) with 999 premutations (performed using metaMDS function from vegan package); (7) the permutational multivariate analysis of variance (PERMANOVA) (performed using adonis2 function with the Bray–Curtis distance metric from the vegan package); (8) ANOVA, followed by a Tukey HSD (Honestly Significant Difference). The statistically significant difference between analyzed groups was considered when the results of the Tukey HSD and ANOVA were less than 0.05. The reported relative abundance of analyzed groups represents the percentage (%) of insects that belong to treated or untreated plots from different locations. Visualization was performed using vegan, ggplot2, and lattice libraries in R with RStudio and Microsoft Excel 2010. 

## 3. Results

### 3.1. Diversity of Epigeal Insects

**Figure 2:**
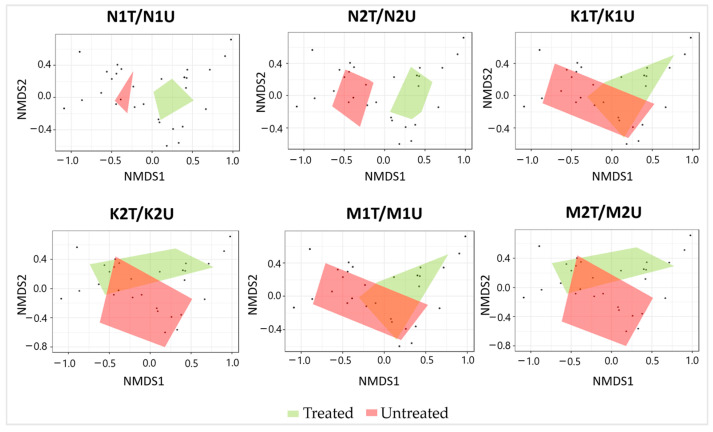
Ordination diagram based on NMDS of epigeal insect assemblages detected in association with treated and untreated *P. sylvestris* plots in different forest stands. Each black dot in the diagrams represents an individual insect species.

During the study period, a total of 7210 individuals (4380 in treated and 2830 in untreated plots) of epigeal insects was trapped (Supplementary Table S1). The relative abundance of the epigeal insects, species richness, Shannon’s index, and NMDS between treated and untreated plots in Kapčiamiestis and Marcinkonys did not differ significantly (*p* > 0.05) in contrast to Neringa (Table 2). The relative abundance of epigeal insects in Neringa was significantly higher (*p* < 0.05) in treated (2274 individuals) than in untreated (703 individuals) plots. However, species richness in Neringa was significantly lower (*p* < 0.005) in treated than in untreated plots. Shannon’s diversity was lower in treated (N1T + N2T) than in untreated (N1U + N2U) plots (*p* < 0.05). The epigeal insect assemblages showed (the PERMANOVA confirmed) significant differences (*p* < 0.05) between treated and untreated plots in both forest stands (N1T/N1U: R = 0.056, *p* < 0.05; N2T/N2U: R = 0.9795, *p* < 0.05) (Figure 2). 

**Figure 3:**
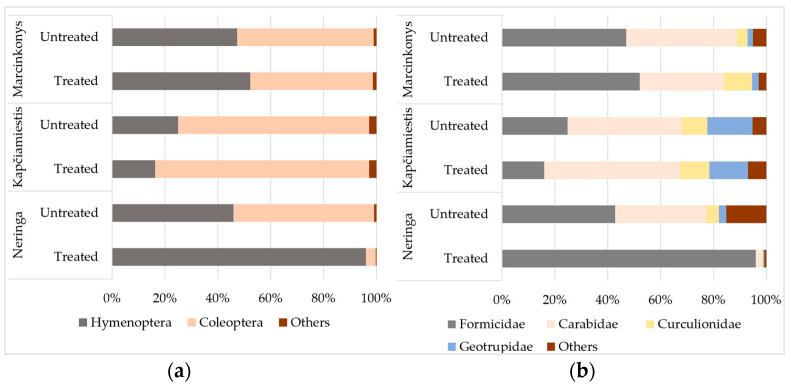
The relative abundance (%) of epigeal insects in treated and untreated *P. sylvestris* plots in different locations, classified by (**a**) order and (**b**) family. Insect orders and families with the relative abundance of less than 2% are marked as “Others”.

In contrast to Neringa, there were only minor variations in the relative abundance of insects in treated and untreated plots in Kapčiamiestis and Marcinkonys (Figure 3). Hymenopterans had significantly higher (*p* < 0.05) relative abundance in treated (96.1%) than in untreated (45.9%) plots in Neringa (Figure 3a), while beetles had higher relative abundance in untreated (53.2%) than in treated (3.6%) plots. The relative abundance of insects from the Formicidae (Hymenoptera) family was 96.0% in treated and 42.8% in untreated plots in Neringa (Figure 3b). The relative abundance of the most frequently detected coleopterans, such as Carabidae, Curculionidae, and Geotrupidae, was 34.4%, 4.8%, and 2.8%, respectively in untreated, and 2.2%, 0.6%, and 0.4%, respectively, in treated plots. The relative abundance of non-target epigeal lepidopterans (*Phalera bucephala* (L.): Notodontidae) was less than 2% (Supplementary Table S1), and they were combined with other less abundant orders, including Diptera, Hemiptera, Dictyoptera, Neuroptera, and Archaeognatha as “Others” (Figure 3).

*Formica rufa* and *Myrmica rubra* were the most dominant insect species in Neringa (Table 3). The relative abundance of *F. rufa* and *M. rubra* in treated plots was 46.2% and 49.3%, respectively, while in untreated plots it was 8.5% and 32.1%. In contrast, the relative abundance of the beetles *Pterostichus niger*, *Calathus micropterus*, and *Staphylinus erythropterus* in Neringa was significantly higher (*p* < 0.05) in untreated (11.1%, 9.0%, and 7.0%, respectively) than in treated (0.2%, 0.4%, and 0.1%, respectively) plots. The relative abundance of *Carabus arcensis* was higher in untreated than in treated plots in Kapčiamiestis (22.9% and 15.5%, respectively) and Marcinkonys (38.9% and 22.2%, respectively).

Among the epigeal insects, entomophagous composed the highest part in each location. The relative abundance of entomophagous insects showed non-significant differences (*p* > 0.05) between treated and untreated plots in Kapčiamiestis and Marcinkonys. In contrast, the relative abundance of entomophagous insects in Neringa was significantly higher (*p* < 0.05) in treated (98.5%) than in untreated (87.9%) plots. There was little variation in the relative abundance of phytophagous, coprophagous, and others, including dendrophagous, mycophagous, necrophagous, polyphagous, and saprophagous, between treated and untreated plots within each location. 

### 3.2. Diversity of Arboreal Insects

**Figure 4:**
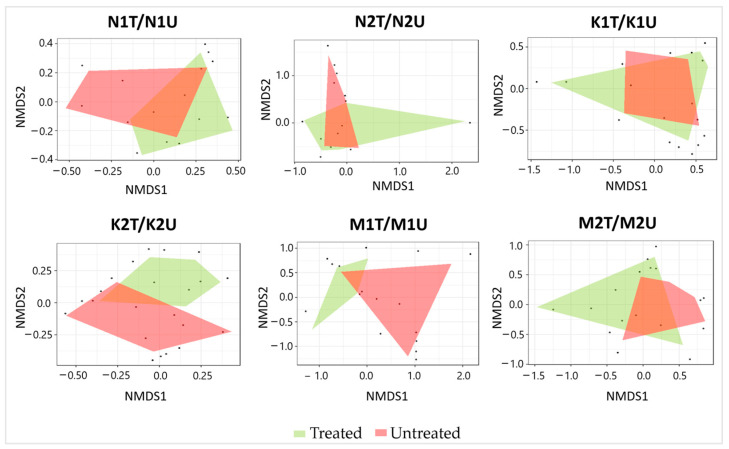
Ordination diagram based on NMDS of arboreal insect assemblages detected in association with treated and untreated *P. sylvestris* plots in different forest stands. Each black dot in the diagram represents an individual insect species.

A total of 380 and 448 arboreal insect individuals were captured in treated and untreated plots, respectively (Supplementary Table S2). The relative abundance of arboreal insects was significantly higher (*p* < 0.05) in untreated than in treated plots at Neringa and Marcinkonys (154 vs. 127 insect individuals, and 127 vs. 82 insect individuals, respectively) (Table 4). Meanwhile, neither the Chi-square test nor Shannon’s diversity showed significant differences (*p* > 0.05) between treated and untreated plots within each location. NMDS of the arboreal insect assemblages showed (and PERMANOVA confirmed) significant differences (*p* < 0.05) between treated and untreated plots in one of the two forest stands within each location (N1T/N1U: R = 0.352; K2T/K2U: R = 0.424; M1T/M1U: R = 0.300) (Figure 4). 

**Figure 5:**
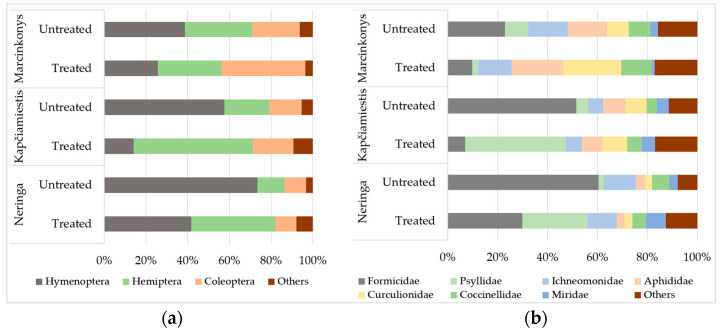
Relative abundance (%) of arboreal insects in treated and untreated *P. sylvestris* plots in different locations classified by (**a**) order and (**b**) family. Insect orders and families with a relative abundance of less than 2% are marked as “Others”.

The relative abundance of hymenopterans was significantly lower (*p* < 0.05) in treated than in untreated plots in Neringa (41.7% and 73.4%), Kapčiamiestis (14.0% and 57.5%), and Marcinkonys (25.6% and 38.6%), respectively (Figure 5a). The decrease of the relative abundance of hymenopterans was mostly influenced by a reduction in ant numbers (Figure 5b). Meanwhile, the relative abundance of hemipterans was higher in treated than in untreated plots in Neringa (40.2% and 13.0%) and Kapčiamiestis (57.3% and 21.6%) (Figure 5a), caused mostly by Psyllidae (Figure 5b). The relative abundance of coleopterans was 40.2% in treated and 22.8% in untreated plots in Marcinkonys (Figure 5a). It was influenced by a higher relative abundance of Curculionidae (23.2% and 8.7%, respectively) (Figure 5b). The orders with relative abundance ≤ 2%, including the non-target geometrid and tortricid Lepidoptera (Supplementary Table S2) and other orders (Diptera, Dictyoptera, Neuroptera, Odonata, Orthoptera, and Psocoptera) are collectively shown as “Others” (Figure 5).

The relative abundance of *Lasius* sp. and *F. rufa* was lower in treated plots in Neringa (7.1% and 19.7%, respectively) and Kapčiamiestis (4.7% and 0.6%, respectively) than in untreated ones (Table 5). No individuals of *F. rufa* were found in Marcinkonys’ treated plots, while the relative abundance of *F. rufa* in untreated plots was 11.0%. Unlike *F. rufa*, the relative abundance of *Strophosomus capitatum* in Marcinkonys was higher in treated than in untreated plots. The relative abundance of Ichneumonidae sp. showed non-significant (*p* > 0.05) differences between treated and untreated plots (*p* > 0.05).

Among arboreal insects, entomophagous and phytophagous ones were the most common at each location. The relative abundance of entomophagous insects was 52.8% in treated and 81.8% in untreated plots in Neringa, 23.4% in treated and 65.3% in untreated plots in Kapčiamiestis, and 39.0% in treated and 52.8% in untreated plots in Marcinkonys.

## 4. Discussion

Previous studies on the potential control of *L. monacha* outbreaks have focused mainly on the use of pheromone traps [46], natural predators [47], entomopathogenic fungi [48] and viruses [49], or the bioinsecticide Foray 76B (*B. thuringiensis* subspecies *kurstaki* (*Btk*)) [50]. However, studies on the indirect effects of the *Btk* treatment on non-target insect populations were lacking. Our results highlight changes in non-target epigeal and arboreal insect diversity in *P. sylvestris* stands after treatment with Foray 76B.

Among all collected non-target insects in the year following mass *L. monacha* outbreaks, the most dominant were Coleoptera, Hymenoptera, and Hemiptera, while non-target Lepidoptera constituted a small part. Shifts in the diversity of these orders could be directly (for Lepidoptera) and indirectly (for Coleoptera, Hymenoptera, and Hemiptera) affected by *Btk* and may act as indicators of forest stress [51,52,53]. For instance, Coleoptera, which are considered the most species-rich order among insects [54], can either help to control populations of other insects [51] or can contribute to nutrient cycling and decomposition processes [55]. The findings of our study revealed that the changes in the diversity of Coleoptera mostly depended on the population of ground beetles (Carabidae), which spend their entire life cycle on the forest floor or underground [56]. Hymenopterans, including bees, wasps, and ants, can also contribute to insect pest control through their functions in predation [57], decomposition [58], support of the food web [59], and parasitism [60]. Our study showed that the diversity of epigeal and arboreal Hymenoptera was similar, highlighting their potential role in facilitating trophic connections between different forest strata [61]. This can be influenced by their life cycle and active vertical movement [62]. Shifts in the diversity of true bugs (Hemiptera) can also indicate changes in forest ecosystems [63]. In the year following mass *L. monacha* outbreaks, hemipterans exhibited higher diversity in the *P. sylvestris* canopy compared to the forest floor, confirming their ecological functions as sapsuckers [64]. Lepidoptera are influential in forest ecosystems, and, as prey, they provide nutrients for entomophagous insects, birds, and bats [65]. Other studies showed that the recovery after *Btk* treatment of non-target Lepidoptera varies among different species [66]. For instance, the populations of non-target Lepidoptera in Douglas-fir forests after *Btk* treatment may partially recover in the following year and require at least two years to fully recover [67]. However, based on the low abundance of Lepidoptera in our study, it is difficult to compare these results with other similar studies.

The results of our study showed an indirect association between the applied sprays with Foray 76B and the species richness of non-target insects in Neringa. The species richness of the epigeal insects was lower in bioinsecticide-treated *P. sylvestris* plots, where the 1st and 2nd instar larvae of the pest are usually killed within 2–5 days after the treatment [68]. However, the species richness of arboreal insects did not differ between treated and untreated *P. sylvestris* plots. Nevertheless, the relative abundance of different orders of non-target insects in tree canopies was clearly distinguished between treated and untreated plots in all locations. A higher relative abundance of arboreal psyllids (sapsuckers) was observed in the bioinsecticide-treated plots. It is known that sapsuckers are indirectly driven by environmental factors that affect the defenses and nutritional quality of trees [69]. Despite being influenced by drought, the general association of sapsuckers with defoliators is more complex [69,70]. Food source competition between sapsuckers and defoliators may lead to the fact that the timing and frequency of their outbreaks differ [71]. Increased relative abundance of sapsuckers in Foray 76B-treated plots could indicate that trees are still under physiological stress after the disruption of their natural enemies—*L. monacha* larvae [72].

The abundance of Formicidae ants could be related to the populations of defoliator [73] and psyllids [74]. We determined a lower population of arboreal red wood ants (*Formica rufa*) in Foray 76B-treated than in untreated *P. sylvestris* stands. This may indicate the role of *F. rufa* in controlling still active defoliator outbreaks in untreated stands. Even though the food-searching behavior of *F. rufa* is primarily related to the abundance of the psyllids, and approximately 85% of the dry mass of the *F. rufa* diet consists of honeydew from them [75,76,77], a higher relative abundance of psyllids did not induce an increased population of *F. rufa* ants in the canopies of treated stands. However, the increased psyllid population might have a stimulating effect on the increased prevalence of the epigeal *F. rufa* ants, which are characterized by their adaptation and ability to navigate through different ecological strata, including the forest canopy, understory, and forest floor, as much as needed [78,79,80,81,82]. Given that *F. rufa* has long been valued for its role in controlling insect pest outbreaks in temperate and boreal forests [83], it is possible that an increase in their arboreal population during the outbreaks of *L. monacha* could naturally contribute to pest management.

The results of our study also indicated significant changes in the diversity of epigeal Coleoptera in the following year after applied treatment with bioinsecticide Foray 76B. The most dominant species of ground beetle was *Carabus arcensis*, which is common in *P. sylvestris* forests in eastern Europe [84]. We found a decreased relative abundance of *C. arcensis* in treated *P. sylvestris* plots, which might be influenced by less direct sunlight on the forest floor through the canopy after defoliation of the trees was stopped [85], or the increased relative abundance of epigeal *F. rufa* ants [83]. Several field studies have reported that the association between the relative abundance of certain species of Carabidae and *F. rufa* is based on their interference competition [86,87]. *C. arsensis* can be a bioindicator of certain biotic and abiotic stress in forests [88]. However, due to climate change and habitat destruction, the species abundance is declining in Europe [89,90]. Our study revealed that treatment with Foray 76B may indirectly contribute to *C. arcensis* decline. Nevertheless, the proliferation of *L. monacha* outbreaks in recent years [25] could have a serious impact on *C. arcensis* populations in Europe.

Sudden fluctuations in the relative abundance of forest insects due to their feeding habits may result in significant damage to overall forest health [91,92]. In our study, the relative abundance of arboreal entomophagous and phytophagous insects distinguished between Foray 76B-treated and untreated *P. sylvestris* stands. A lower ratio of the arboreal entomophagous in treated plots in the following year after the outbreaks could be influenced by the reduced level of the defoliator [92]. However, an increased relative abundance of arboreal phytophagous insects in treated plots may be related to several factors: (1) better nutritional quality of tree needles and (2) *P. sylvestris* trees still experiencing stress caused by the outbreaks of *L. monacha*.

Overall, the results of the conducted studies show that treatment with Foray 76B to control the mass outbreaks of *L. monacha* may indirectly influence the diversity and species composition of non-target insect assemblages, resulting in a possible impact on the health and resilience of the forest stands. The study provides valuable knowledge regarding the interactions between non-target insects and how they are affected by biotic disturbance. Further research to assess whether changes in the diversity and composition of non-target insect assemblages are a long-term phenomenon, or whether resilience may occur over time, is needed. Although the short-term effect of Foray 76B on non-target organisms might be negligible, the impact of frequent repeated applications of biological agents on most ecosystems is not well known [32,34,93]. It is probable that any regular disruption of insect assemblages, due to chemical or microbial insecticides or natural factors, could have long-term deleterious effects on ecosystem structure [94]. Therefore, complex research, including other ecosystem factors such as soil chemistry and microbial diversity after the applied treatment, is demanding. 

## Figures and Tables

**Table 1:** Characteristics of treated and untreated *P. sylvestris* forest stands. Data obtained by permission from the State Forest Cadaster as of 2021.

Location	Sampling Plot Code *	Latitude (N)	Longitude (E)	Age (y)	Mean Height (m)	Mean Diameter (cm)	Forest Site Type **	Forest Vegetation Type ***
Neringa	N1T	55°41′47.6″	21°06′22.1″	120	16.5	23.0	Nal	cl
	N1U	55°40′38.8″	21°06′24.5″	65	16.7	18.0	Nal	cl
	N2T	55°19′54.0″	21°02′29.0″	60	18.1	19.0	Nal	cl
	N2U	55°24′30.2″	21°04′42.1″	110	11.5	13.0	Nal	cl
Kapčiamiestis	K1T	54°02′41.0″	23°32′16.6″	89	29.3	34.0	Nbl	vm
	K1U	54°02′09.8″	23°32′08.6″	74	23.6	28.7	Nbl	vm
	K2T	54°01′07.3″	23°31′32.2″	89	25.1	29.9	Nbl	vm
	K2U	54°00′16.8″	23°30′05.6″	76	28.2	31.3	Nbl	vm
Marcinkonys	M1T	54°06′23.8″	24°25′58.8″	98	28.6	35.4	Nbl	vm
	M1U	54°07′33.5″	24°27′17.1″	78	25.2	25.6	Nbl	vm
	M2T	54°01′51.8″	24°26′06.3″	125	25.3	25.7	Nbl	vm
	M2U	54°02′09.5″	24°25′26.7″	83	26.8	29.6	Nal	cl

* Different letters at the end of the sampling plot code indicate the type of plot, i.e., T—treated, U—untreated. ** N: Normal humidity, a: very poor fertility, b: poor fertility, l: light soil texture. *** cl: cladoniosum, vm: vaccinio-myrtilliosum [38].

**Table 2:** Total number, relative abundance (%), number of insect species, and Shannon diversity of trapped epigeal insects in treated and untreated *P. sylvestris* plots in different forest stands.

Location	Sampling Plot Code *	Total No. of Insects	Relative Abundance, %	No. of Insect Species	Shannon Diversity
Neringa	N1T	1021	14.2	25	0.43
	N2T	1253	17.4	23	0.56
	N1T + N2T	2274	31.5	36	0.99
	N1U	442	6.1	37	2.66
	N2U	261	3.6	17	1.65
	N1U + N2U	703	9.8	41	2.43
	Total	2977	41.3	51	1.56
Kapčiamiestis	K1T	464	6.4	31	2.35
	K2T	582	8.1	37	2.71
	K1T + K2T	1046	14.5	45	2.75
	K1U	472	6.6	30	2.33
	K2U	461	6.4	33	2.48
	K1U + K2U	933	12.9	41	2.49
	Total	1979	27.4	56	2.71
Marcinkonys	M1T	590	8.2	25	1.54
	M2T	470	6.5	29	2.08
	M1T + M2T	1060	14.7	40	1.87
	M1U	973	13.5	31	1.39
	M2U	221	3.1	26	1.90
	M1U + M2U	1194	16.6	40	1.65
	Total	2254	31.3	51	1.79

* Different letters at the end of the sampling plot code indicate the type of plot, i.e., T—treated, U—untreated.

**Table 3:** Relative abundance (%) of the 15 most abundant epigeal insect species in treated and untreated *P. sylvestris* plots in different locations, organized alphabetically by species name.

Species	Order/Family	Relative Abundance, %								
		Neringa			Kapčiamiestis			Marcinkonys		
		* N1T + N2T	N1U + N2U	Neringa, Total	K1T + K2T	K1U + K2U	Kapčiamiestis, Total	M1T + M2T	M1U + M2U	Marcinkonys, Total
*Calathus errathus* Sahlbg.	Coleoptera/ Carabidae	0.2	2.4	0.7	6.1	1.6	4.0	0.5	0.1	0.3
*Calathus micropterus* Duftschmid.	Coleoptera/ Carabidae	0.4	9.0	2.5	5.7	6.2	6.0	4.2	0.5	2.3
*Carabus arcensis* Hbst.	Coleoptera/ Carabidae	0.2	1.7	0.6	15.5	22.9	19.0	22.2	38.9	31.0
*Carabus violeaceus* L.	Coleoptera/ Carabidae	0.2	3.4	0.9	4.8	1.9	3.4	0.8	1.1	0.9
*Formica rufa* L.	Hymenoptera/ Formicidae	46.2	8.5	37.3	3.9	15.9	9.6	46.7	40.7	43.5
*Formicidae* sp.	Hymenoptera/ Formicidae	0.6	2.1	0.9	1.5	0.4	1.0	0.0	0.0	0.0
*Geotrupes stercorosus* Scriba.	Coleoptera/ Geotrupidae	0.3	0.3	0.3	14.3	16.2	15.2	1.7	1.6	1.6
*Geotrupes vernalis* L.	Coleoptera/ Geotrupidae	0.1	2.6	0.7	0.3	0.9	0.6	0.8	0.4	0.6
*Hylobius abietis* L.	Coleoptera/ Curculionidae	0.2	2.7	0.8	2.0	1.2	1.6	5.1	1.2	3.0
*Myrmica rubra* L.	Hymenoptera/ Formicidae	49.3	32.1	45.2	10.5	8.6	9.6	5.5	6.3	5.9
*Pterostichus aterrimus* Herbst.	Coleoptera/ Carabidae	0.0	0.1	0.0	3.3	0.8	2.1	0.0	0.0	0.0
*Pterostichus niger* Schaller.	Coleoptera/ Carabidae	0.2	11.1	2.8	9.6	5.4	7.6	1.7	0.5	1.1
*Pterostichus oblongopunctatum* F.	Coleoptera/ Carabidae	0.1	0.7	0.2	4.4	1.8	3.2	1.4	0.3	0.8
*Staphylinus erythropterus* L.	Coleoptera/ Staphylinidae	0.1	7.0	1.7	1.2	0.1	0.7	0.2	0.1	0.1
*Strophosomus capitatum* De Geer.	Coleoptera/ Curculionidae	0.3	1.3	0.5	8.9	7.6	8.3	4.7	2.2	3.4
Total of 15 species		98.2	85.1	95.1	92.1	91.4	91.8	95.5	93.8	94.6

* Different letters at the end of the sampling plot code indicate the type of plot, i.e., T—treated, U—untreated.

**Table 4:** Total number, relative abundance (%), number of insect species, and Shannon diversity of arboreal insects in treated and untreated *P. sylvestris* plots in different forest stands.

Location	Sampling Plot Code *	Total No. of Insects	Relative Abundance, %	No. of Insect Species	Shannon Diversity
Neringa	N1T	93	11.2	16	1.96
	N2T	34	4.1	13	2.26
	N1T + N2T	127	15.3	24	2.45
	N1U	71	8.6	15	2.12
	N2U	83	10.0	14	1.89
	N1U + N2U	154	18.6	22	2.25
	Total	281	33.9	33	2.54
Kapčiamiestis	K1T	57	6.9	17	2.56
	K2T	114	13.8	25	1.96
	K1T + K2T	171	20.7	33	2.49
	K1U	52	6.3	19	2.60
	K2U	115	13.9	21	2.06
	K1U + K2U	167	20.2	29	2.47
	Total	338	40.8	41	2.75
Marcinkonys	M1T	39	4.7	15	2.26
	M2T	43	5.2	14	2.04
	M1T + M2T	82	9.9	24	2.53
	M1U	77	9.3	18	2.47
	M2U	50	6.0	20	2.67
	M1U + M2U	127	15.3	28	2.75
	Total	209	25.3	39	2.87

* Different letters at the end of the sampling plot code indicate the type of plot, i.e., T—treated, U—untreated.

**Table 5:** The relative abundance (%) of the 15 most abundant arboreal insect species in treated and untreated *P. sylvestris* plots in different locations, organized alphabetically by species name.

Species	Order/Family	Relative Abundance, %								
		Neringa			Kapčiamiestis			Marcinkonys		
		* N1T + N2T	N1U + N2U	Neringa, Total	K1T + K2T	K1U + K2U	Kapčiamiestis, Total	M1T + M2T	M1U + M2U	Marcinkonys, total
*Adalia bipunctata* L.	Coleoptera/ Coccinellidae	1.6	5.8	3.9	2.9	0.6	1.8	3.7	0.0	1.4
*Aleyrodidae* sp.	Hemiptera/ Aleyrodidae	0.0	0.6	0.4	1.2	1.8	1.5	3.7	0.0	1.4
*Barbitistes* *constrictus* Br.	Orthoptera/ Phaneropteridae	0.8	0.0	0.4	4.1	0.6	2.4	0.0	0.0	0.0
*Brachonyx* *pineti* Payk.	Coleoptera/ Curculionidae	0.0	0.6	0.4	4.1	0.6	2.4	0.0	0.0	0.0
*Cinara sp.*	Hemiptera/ Aphididae	3.1	3.9	3.6	8.2	9.0	8.6	20.7	15.7	17.7
*Formica rufa* L.	Hymenoptera/ Formicidae	19.7	28.6	24.6	0.6	11.4	5.9	0.0	11.0	6.7
*Ichneuomonidae* sp.	Hymenoptera/ Ichneuomonidae	11.8	13.0	12.5	6.4	6.0	6.2	13.4	15.7	14.8
*Lasius* sp.	Hymenoptera/ Formicidae	7.1	18.2	13.2	4.7	34.7	19.5	4.9	5.5	5.3
*Miridae* sp.	Hemiptera/ Miridae	5.5	0.0	2.5	3.5	1.2	2.4	0.0	2.4	1.4
*Myrmica rubra* L.	Hymenoptera/ Formicidae	3.1	13.6	8.9	1.8	5.4	3.6	4.9	6.3	5.7
*Psyllidae* sp.	Hemiptera/ Psyllidae	26.0	1.9	12.8	40.4	4.8	22.8	2.4	9.4	6.7
*Scymnus* *suturalis Thunb.*	Coleoptera/ Coccinellidae	3.9	0.0	1.8	0.6	1.2	0.9	2.4	7.1	5.3
*Stenodema* *laevigata* L.	Hemiptera/ Miridae	2.4	1.3	1.8	1.2	3.0	2.1	1.2	0.8	1.0
*Strophosomus capitatum* De Geer.	Coleoptera/ Curculionidae	3.1	1.3	2.1	5.3	6.0	5.6	23.2	5.5	12.4
*Tachinidae* sp.	Diptera/ Tachnidae	0.0	0.0	0.0	1.2	3.0	2.1	0.0	0.8	0.5
Total of 15 species		98.2	85.1	95.1	92.1	91.4	91.8	95.5	93.8	94.6

* Different letters at the end of the sampling plot code indicate the type of plot, i.e., T—treated, U—untreated.

## Data Availability

The relative abundance (%) of epigeal and arboreal insects in *Pinus sylvestris* in Lithuania presented in this study are presented in Tables S1 and S2. Other data related to the study are available on request from the corresponding author.

## Supplementary Materials

The following supporting information can be downloaded at: https://www.mdpi.com/article/10.3390/insects15030200/s1, Supplementary Table S1: Relative abundance (%) of epigeal insects in *Pinus sylvestris* in Lithuania. Supplementary Table S2: Relative abundance (%) of arboreal insects in *Pinus sylvestris* in Lithuania.
